# Editorial

**Published:** 1904-04-15

**Authors:** 


### EDITORIAL.

CHCAGO DENTAL COLLEGE AND UNIVERSITY OF ILLINOIS CONSOLIDATION FAILS, The agreement whereby the Univesity of Illinois was to take over the Chicago Dental College, and consolidate it with its dental department, has been declared illegal by the courts on the complaint of one of the stockholders of the Chicago Dental College, Dr. Copeland, the Professor of Anatomy, who claimed that the agreement did not have the unanimous consent of the stockholders. It is too bad that this arrangement could not have been consummated, as it would undoubtedly work to the best interest of dental education in the future. It probably would not materially increase the efficiency of this school at once but it would have made the future of the school's policy certain, and give it an educational standing that would be invaluable in maintaining professional education standards in this part of the country. We trust the matter may be readjusted on a legal and satisfactory basis.

—✦—

DENTISTRY A SPECIALTY OF MEDICINE. The Chicago Dental and Chicago Medical Societies have tried to discuss to a practical issue the following resolution: "Resolved, that dentistry is, and should be, regarded as a specialty of medicine. The aim of the medical people was a kindly one, and was to test the spirit of the dental fraternity, to see if it was not possible to find some way in which dentistry might not be considered a specialty of medicine. Of course, the independent ideals that have developed with the profession could not be smothered even in a friendly discussion. The resolution was discussed at great length, and was finally adopted with the following amendments: "That to be fitted to practice it, requires special training in dental colleges." In the discussion no sufficient reasons were given for the consolidation, but many arguments, most of them not new, were made for an independent profession.

If all the concessions demanded are made a part of any compact it had much better not be made. It is true that the relations of medical and dental practitioners often are intimate, and to a certain extent educational lines parallel. But the technical part of the dentist's training and practice is so distinct from the physician's, that there never can be the same intimate associations as necessarily exists between the physician, general surgeon, ophthalmologist and other medical specialists. The time may come when dental practice so changes, and dental education is so changed, that the dentist becomes a medically-trained man, that the two professions may be united, but it can not be so under existing circumstances.

—✦—

Medical and Dental Training for Practice. In the discussion of the resolution that dentistry is a specialty of medicine in the Chicago Dental Society, a prominent physician is reported as saying that the dentist as graduated to-day is a better trained man for the work he has to do than the graduate of the present medical college for the work he is to do. This statement we believe to be true, and there are good reasons why it is so. In medical training a student previous to graduation has little or no opportunity for applying any practical knowledge he may be accumulating. His entire time is spent in class and text-work, and in witnessing clinics and is seldom given an opportunity to put into practice the theories taught. He therefore, loses one of the best methods of cultivating his art. The dental student is thrown on his own resources a great part of his time, but he has the feeling always that there is a responsibility back of him to which he can appeal in time of need. This gradually creates interest and individual responsibility, which gives him a large advantage in beginning practice. And yet we have often heard dentists who had been in practice a year or so make the remark that they had learned more in the year they had been in practice than they had during their whole three years of practice. They failed to see that it was the long three years of application to fundamentals, and even technical details, which made it possible for them to learn so much in a short time. They never could have done it without the mental culture of those three years. In the same way the medical man who gets his training in a good school, though it be lacking in clinical opportunities, will learn faster and more than the medical man trained in an inferior school, or none at all.

---

HONORING OLD GRADUATES. February 27th more than one hundred dentists from various parts of the country assembled in Philadelphia to pay a tribute to the five living graduates of the Philadelphia Dental College of 1854. A kind of semi-centennial celebration as it were. The men, some of them at least, have given largely to the development of our profession, and deserve the encomiums put upon them for their unselfish devotion to the higher interests of the profession. These men were particularly fortunate in having their training under men who held high ideals for the profession, and believed that dentistry was destined to become one of the learned professions. They taught it so faithfully that they made deep impressions on the characters of their students, while teaching technical details. It is a good thing to celebrate such occasions when so much meaning can be put into the festivities. It is a grand thing to cultivate sentiment and honor men who stand for high principles, and we trust every occasion that offers may be utilized. Something is needed to counteract the commercial influence which presses so strongly on the young men in the profession.

Commercial success is not to be discredited, but professional success is ideal and infinitely more desirable to the profession as well as the individual.

